# Photoactive titanium dioxide nanoparticles modify heterotrophic microbial functioning

**DOI:** 10.1007/s11356-021-14090-3

**Published:** 2021-05-02

**Authors:** Mirco Bundschuh, Jochen P. Zubrod, Marco Konschak, Patrick Baudy, Bianca Frombold, Ralf Schulz

**Affiliations:** 1 iES Landau, Institute for Environmental Sciences, University of Koblenz-Landau, Fortstraße 7, D-76829 Landau, Germany; 2 Department of Aquatic Sciences and Assessment, Swedish University of Agricultural Sciences, Lennart Hjelms väg 9, SWE-75007 Uppsala, Sweden; 3 Eußerthal Ecosystem Research Station, University of Koblenz-Landau, Birkenthalstraße 13, D-76857, Eußerthal, Germany

**Keywords:** Nanomaterials, Semi-conductor, Trophic interaction, Ecological effects, Food selection

## Abstract

Nanoparticulate titanium dioxide (nTiO_2_) is frequently applied, raising concerns about potential side effects on the environment. While various studies have assessed structural effects in aquatic model ecosystems, its impact on ecosystem functions provided by microbial communities (biofilms) is not well understood. This is all the more the case when considering additional stressors, such as UV irradiation — a factor known to amplify nTiO_2_-induced toxicity. Using pairwise comparisons, we assessed the impact of UV (UV-A = 1.6 W/m^2^; UV-B = 0.7 W/m^2^) at 0, 20 or 2000 μg nTiO_2_/L on two ecosystem functions provided by leaf-associated biofilms: while leaf litter conditioning, important for detritivorous invertebrate nutrition, seems unaffected, microbial leaf decomposition was stimulated (up to 25%) by UV, with effect sizes being higher in the presence of nTiO_2_. Although stoichiometric and microbial analyses did not allow for uncovering the underlying mechanism, it seems plausible that the combination of a shift in biofilm community composition and activity together with photodegradation as well as the formation of reactive oxygen species triggered changes in leaf litter decomposition. The present study implies that the multiple functions a microbial community performs are not equally sensitive. Consequently, relying on one of the many functions realized by the same microbial community may be misleading for environmental management.

## Introduction

Engineered nanoparticles (NPs) feature unique physicochemical properties (e.g. size, surface area, surface reactivity, charge, shape) relative to their bulk or ionic counterparts (Bundschuh et al. 2016), which makes them suitable for various applications. Nanoparticulate titanium dioxide (nTiO_2_), for instance, is used in a broad range of products including textiles, sunscreens, and facade paints (e.g. Windler et al. 2012), which are partly doped to increase their functionality (Milosevic et al. 2017; Milosevic et al. 2018). Consequently, nTiO_2_ is inevitably released into aquatic ecosystems from point (e.g. wastewater treatment plant effluents; Kiser et al. 2009) and non-point (e.g. from sunscreens during swimming; Gondikas et al. 2014) sources. Its environmental concentrations have been predicted to be in the microgram per litre range (Gottschalk et al. 2013) and were reported to be up to 27 μg/L during the bathing season in a recreational lake in Austria (Gondikas et al. 2014). Although most studies assessing acute or chronic effects indicate only a low risk of nTiO_2_ for aquatic life (e.g. Seitz et al. 2014; Zhu et al. 2010), experimental evidence points towards substantial cross-generational implications in aquatic key species at field-relevant concentrations (Bundschuh et al. 2012).

Moreover, the interaction of nTiO_2_ with environmental variables, in particular ultraviolet (UV) light, can disproportionally increase the ecotoxicological potential of these particles (Jovanovic 2015), which is likely driven by the photocatalytic formation of reactive oxygen species (ROS) at ambient UV intensities (Schaumann et al. 2015). ROS can impair biological systems by initiating oxidative stress through, for instance, damaging polyunsaturated fatty acids within cell membranes (Cabiscol et al. 2000), which negatively affects aquatic organisms (Dalai et al. 2012). However, little is known about how nTiO_2_ in combination with UV affect microbial biofilms that determined carbon and nutrient cycling in many ecosystems and, thus, form the basis of food webs. Nonetheless, it was shown that biofilm communities associated with hard substrate may shift towards ROS-tolerant species with implications in their biomass, metabolic activity (Binh et al. 2016; Wright et al. 2018), and the production of extracellular polymeric substances (Kumari et al. 2014) under combined exposures to nTiO_2_ and UV. Moreover, heterotrophic biofilms, which are often associated with detritus, exhibit an altered enzymatic activity (Schug et al. 2014) under a combined exposure to nTiO_2_ and UV, which points towards effects on ecosystem-level processes such as leaf litter decomposition. While the impact of nTiO_2_ alone on leaf litter decomposition is documented elsewhere (Du et al. 2018; Jain et al. 2019), only one study assessed the combined effect of nTiO_2_ and natural sunlight with unknown UV intensity. In this study, Al Riyami et al. (2019) observed that sunlight mitigates the impact of nTiO_2_ observed in darkness on microbial leaf mass loss, which the authors related to the release of ROS degrading structural polysaccharides in the leaves. Besides leaf decomposition, leaf-associated heterotrophic biofilms — in particular bacteria and fungi — increase leaves’ palatability and nutritional value for leaf-shredding macroinvertebrates (Bärlocher 1985). These biofilms are thus of paramount importance for the integration of energy bound by leaves into stream food webs (Taylor and Chauvet 2014). Given the documented joint impact of nTiO_2_ and UV on bacterial community composition (Binh et al. 2016) and microbial enzyme activity (Schug et al. 2014), an effect in the nutrition of higher trophic levels may be expected as such implications were shown, for instance, for fungicides and antibiotics (Bundschuh et al. 2009; Bundschuh et al. 2011).

To address these knowledge gaps, we investigated the impact of three levels of nTiO_2_ in the absence and presence of ambient UV light on the two microbial functions of leaf decomposition as well as palatability. The three nTiO_2_ concentrations of 0, 20, and 2000 μg/L reflected a control, a field-relevant (Gondikas et al. 2014) and an overdose scenario, respectively. The UV intensity (UV-A = 1.6 W/m^2^; UV-B = 0.7 W/m^2^) applied during the course of this study is well within the range of field-relevant levels (Kalčíková et al. 2014) and at least an order of magnitude below peak intensities measured in Central Europe (Häder et al. 2007). While implications on microbial leaf decomposition were studied by quantifying the microbially mediated leaf mass loss, leaf palatability was assessed via the food choice of the highly selectively feeding amphipod *Gammarus fossarum* Koch (Arsuffi and Suberkropp 1989), a key shredder in many European low-order streams (Dangles et al. 2004). We hypothesized that the presence of UV light would increase the concentration-dependent effects of nTiO_2_ on both functional variables, while the direction of effects might be different in these functional variables as a consequence of changes in the microbial trait composition. Although it was beyond the scope of the present study to characterize changes in the microbial trait composition, we assessed microbial sum parameters and leaf stoichiometry as variables approximating nutritional quality and biofilm characteristics.

## Material and methods

### Nanoparticle preparation

P25, which consists of anatase and rutile crystalline forms (ratio ~75:25; AEROXIDE^®^ TiO_2_ P25; Evonik, Germany) served as model nTiO_2_. The nanoparticles are, according to the producer, 21 nm in size. An additive-free suspension with a concentration of 80 g nTiO_2_/L was provided by the Institute for Particle Technology (TU Braunschweig, Germany), which was further diluted in deionized water to a nominal concentration of 0.02 and 2.00 g nTiO_2_/L. These stock suspensions were pH stabilized (~3.25) using 2 M HCl. The respective mean particle sizes of the two stock suspensions were 81.4 ± 4.3 and 92.6 ± 1.3 nm (*n* = 3) (Delsa™ Nano Submicron Particle Size and Zeta Potential, Beckman Coulter, USA). To ensure a homogenous distribution of nanoparticles in the stock suspensions, the suspension was sonicated for 10 min before test initiation. Subsequently, the stock suspensions were further diluted in the nutrient medium (pH of 7) used for leaf conditioning (Dang et al. 2005) to nominal test concentrations of 0, 20, and 2000 μg nTiO_2_/L. Water samples for the verification of nTiO_2_ exposure were taken at test initiation and analysed by ICP-MS (inductively coupled plasma quadrupole mass spectrometry; XSeries II, Thermo Fisher Scientific, Germany). Details are provided elsewhere (Rosenfeldt et al. 2014). Measured concentrations deviated no more than 20% from the nominal concentration, justifying the use of the latter throughout the document. Particle size distribution was not monitored during the test duration in the nutrient medium, but we expect the particle size to increase soon after spiking due to both the ion strength and pH of the medium, with the latter deviating substantially from the point of zero charge.

### Sampling of leaves, microorganisms, and gammarids

Sampling procedures are described in detail elsewhere (Zubrod et al. 2015b). Consequently, we highlight here the principles only: *Alnus glutinosa* leaves (black alder) were picked during leaf fall in 2014 (49° 11′ N, 8° 05′ E) and stored frozen. Microbial inoculum was generated by deploying 500 alder leaves for 14 days in a near-natural stream (49° 33′ N, 8° 02′ E). Subsequently, these field conditioned leaves were mixed with another 500 unconditioned leaves and cultured for another 14 days in total darkness at 16 ± 1°C in the conditioning medium.

Cryptic lineage B of *G. fossarum* (Feckler et al. 2014) was kick-sampled from another stream (49° 14′ N, 8° 03′ E). For the experiment, adult males 6 to 8 mm in body length and visually free from acanthocephalan parasites were used. During the 7-day acclimation to SAM-5S medium (detailed in Borgmann 1996), gammarids were fed with microbially conditioned *Alnus* leaves. To level their appetite, gammarids were starved for a few days before being used in the experiment.

### Main experiment

The experiment comprised six treatments: each nTiO_2_ concentration (0, 20, and 2000 μg/L) was assessed at UV-A and UV-B intensities of approximately 1.6 and 0.7 W/m^2^ (lamp: Heraeus Magic Sun 23/160 R 160 W), respectively, or in darkness. The employed UV levels can be considered field-relevant as intensities of 6.5 and 0.3 W/m^2^ have been measured during a cloudy mid-summer day at our university campus (Kalčíková et al. 2014).

The experiment followed in principle Bundschuh et al. (2009) with some alterations. For the quantification of potential implications in microorganism-mediated leaf decomposition and leaf palatability as indicated through gammarids’ food choice, four leaf discs were cut from the same defrosted *Alnus* leaves. Discs were dried to a constant weight (60°C for 24 h) and weighed to the nearest 0.01 mg. Two discs cut from the same leaf were microbially conditioned in one of the six treatments and the remaining two in another treatment using a pairwise design. In total, seven pairwise tests were performed where either the impact of UV irradiation at each of the nTiO_2_ concentrations was targeted or the role of the nTiO_2_ concentration nested in the respective UV level (i.e. presence or absence). For this purpose, the discs were placed in glass aquaria (seven replicates per treatment) accompanied by leaves supporting the assessment of stoichiometry and microbial parameters (see below). Replicates contained 4 L constantly stirred and aerated nutrient medium (Dang et al. 2005) with the respective nTiO_2_ concentration and leaf inoculum (10 g fresh weight). Conditioning took place at 16 ± 1°C either in darkness or under UV light (day:night = 12 h:12 h). The medium was renewed every third day.

Following microbial conditioning (12 days), leaf discs were first rinsed in clean SAM-5S medium for half an hour. Two leaf discs microbially conditioned in two treatments but cut from the same leaf were offered gammarids to assess their food choice. For this purpose, a 300-mL crystallization dish with 100 mL of SAM-5S medium was used and feeding was allowed for 24 h (at 16 ± 1°C in darkness). The two remaining discs from the same leaf were protected from gammarid feeding in the same dish serving the quantification of microbial leaf decomposition (Bundschuh et al. 2009; Zubrod et al. 2015a). Moreover, microbial leaf mass loss was considered during the calculation of gammarid leaf consumption (Bundschuh et al. 2009; Zubrod et al. 2015a). At the termination of each food choice experiment, gammarids and remaining leaf material were dried and weighed as described above. Of the 49 replicates per food choice assay, those with dead or moulting gammarids were not considered during statistical analyses of their behaviour, reducing the replication in some situations to 39.

### Microbial and stoichiometric properties of leaves

Leaf-associated microbes were characterized on discs conditioned in the same aquaria as those used to quantify the functional endpoints, leading to seven replicates. Ergosterol served as proxy for fungal biomass (Gessner 2005) and was quantified by HPLC (high-performance liquid chromatography, 1200 Series, Agilent Technologies, USA) following solid-phase extraction. The bacterial cells were detached from the leaf surface by ultrasound, stained with SYBR Green II (Molecular Probes, USA) and counted under an epifluorescence microscope. The cell counts were finally normalized to the leaf dry mass as detailed in Buesing (2005).

The elemental stoichiometry (carbon, hydrogen, nitrogen, and sulfur) of leaves from the different treatments was measured. Therefore, dried and ground leaf material (2–5 mg) was weighed into an aluminium weighing boat to the nearest 0.0001 mg (SE 2-OCE scale, Mettler Toledo GmbH, Germany) and analysed (MICRO cube CHNS Analyzer, Elementar Analysensysteme GmbH, Germany).

### Statistics

Data were visually checked for normality. Levene’s test was used to assess homogeneity of variances. Statistically significant differences between treatments for paired datasets, namely the functional response variables, were assessed using paired *t*-tests or Wilcoxon signed-rank tests as a nonparametric alternative. For the remaining, unpaired data (i.e. fungal biomass, bacterial density, and CHNS data), the significance of both studied variables (i.e. nTiO_2_ exposure and UV irradiation) was assessed using two-way analysis of variance (ANOVA) based on either original or rank-transformed values.

## Results and discussion

### Food selection by gammarids

**Fig. 1:**
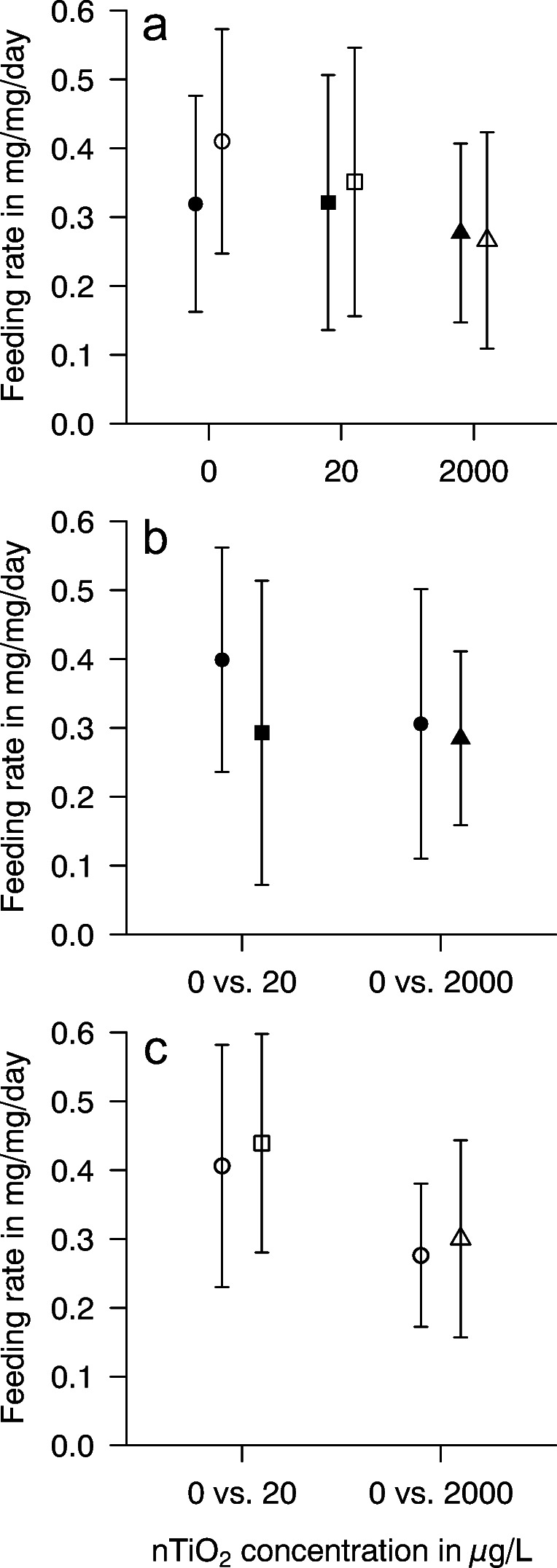
Mean (±95% confidence interval (CI)) leaf mass consumed by *G. fossarum* in food choice experiments. **a** The impact of UV at a given nTiO_2_ concentration was assessed: Gammarids had the choice between leaf discs conditioned in darkness (filled symbols) or under UV irradiation (open symbols) in combination with 0 (circles), 20 (squares), and 2000 (triangles) μg nTiO_2_/L, respectively (pairwise *t*-tests; *p* > 0.5; *n* ≥ 39). **b** The impact of two nTiO_2_ concentrations during conditioning in darkness was assessed: gammarids had the choice between leaf discs conditioned in darkness (filled symbols) in combination with 0 (circles) vs 20 (square) and 2000 (triangle) μg nTiO_2_/L, respectively (pairwise *t*-tests; *p* > 0.5; *n* ≥ 39). **c** The impact of two nTiO_2_ concentrations during conditioning under UV irradiation was assessed: gammarids had the choice between leaf discs conditioned under UV irradiation (open symbols) in combination with 0 (circles) vs 20 (square) and 2000 (triangle) μg nTiO_2_/L, respectively (pairwise *t*-tests; *p* > 0.5; *n* ≥ 39)

**Fig. 2:**
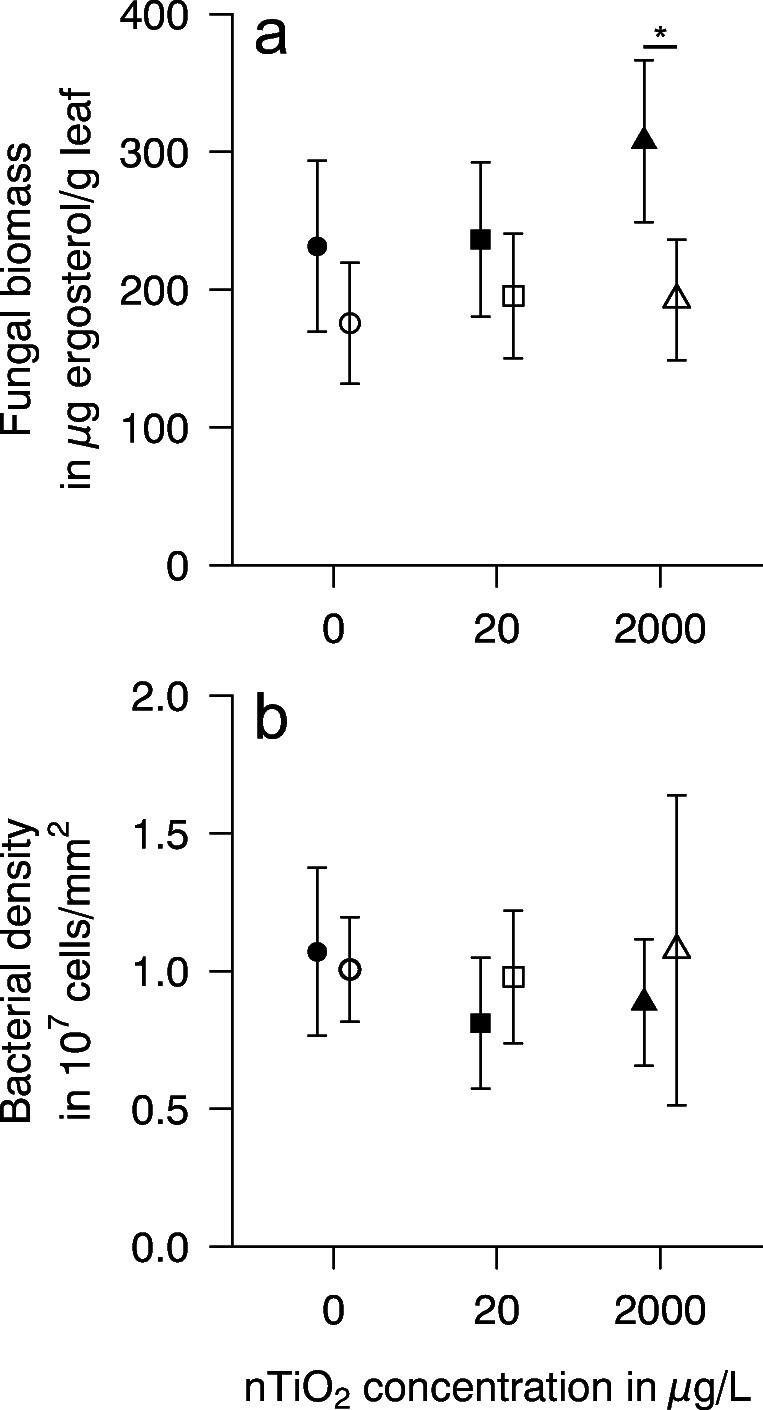
Mean (±95% CI) **a** fungal biomass measured as ergosterol and **b** bacterial density after 12 days of microbial conditioning in darkness (filled symbols) or under UV irradiation (open symbols) fully crossed with 0 (circles), 20 (squares), and 2000 (triangles) μg nTiO_2_/L, respectively (pairwise *t*-tests; *n* = 7; asterisk indicates a significant difference at *p* < 0.001)

Just as a range of other chemicals of anthropogenic origin, NPs interfere with aquatic life at various levels of ecological complexity (e.g. Bundschuh et al. 2018). While the number of studies targeting NP-induced effects on biofilms shaping autotrophic and heterotrophic food webs bottom-up is increasing, the interaction of these NPs with additional factors influencing their fate and effect is still rather limited. In a first step, we assessed the effects of nTiO_2_ on leaf palatability for shredders, hypothesizing that UV irradiation would negatively influence leaf palatability for shredders, which could be explained by shifts in microbial conditioning. Pairwise comparisons indicate that gammarids consumed nearly equal amounts of leaf material conditioned in absence relative to the presence of UV. The observation was independent of the nTiO_2_ concentration present during conditioning (Fig. 1a), speaking against our hypothesis. This lack of a clear feeding preference by *G. fossarum*, a highly selective species (Arsuffi and Suberkropp 1989), suggests that there is no substantial difference in microbial and stoichiometric variables. While this assumption holds for most variables (Fig. 2, Table 1), fungal biomass was at each nTiO_2_ concentration higher in darkness relative to the presence of UV light, leading to a significant UV effect (Table 2). This difference was with an effect size (=magnitude of effect) of 60% significant at the highest test concentration, that is 2000 μg nTiO_2_/L (*p* = 0.007, *n* = 7) (Fig. 2a). As fungi are assumed to trigger food selection and a higher fungal biomass was often linked to a higher palatability for shredders (Fourcreau et al. 2013), our data point to a shift in leaf-associated fungal communities that was, despite a higher biomass, similarly attractive. There is, however, no community composition data available from our present work supporting this assumption.

**Table 1:** Mean percentage (with range) of carbon (C), hydrogen (H), nitrogen (N), and sulfur (S) contained in leaf material microbially conditioned in the presence of different nTiO_2_ concentrations in the absence or presence of ambient UV irradiation (UV-A = 1.6 W/m^2^; UV-B = 0.7 W/m^2^) for 12 days

nTiO_2_	UV	C (%)	H (%)	N (%)	S (%)
0 μg/L	No	49.10 (48.07–50.12)	6.16 (6.01–6.30)	5.82 (5.45–6.20)	0.52 (0.43–0.61)
	Yes	49.80 (48.69–50.91)	6.29 (6.10–6.49)	5.82 (5.53–6.11)	0.39 (0.23–0.55)
20 μg/L	No	49.22 (48.14–50.30)	6.21 (6.09–6.32)	5.85 (5.46–6.23)	0.44 (0.35–0.52)
	Yes	50.01 (48.98–51.05)	6.22 (6.15–6.30)	5.83 (5.57–6.09)	0.46 (0.35–0.58)
2000 μg/L	No	49.46 (48.40–50.52)	6.12 (5.88–6.35)	5.92 (5.60–6.23)	0.43 (0.25–0.61)
	Yes	49.61 (48.79–50.44)	6.25 (6.08–6.41)	5.71 (5.38–6.03)	0.37 (0.20–0.53)

**Table 2:** Output table of the two-factorial ANOVAs performed on untransformed or rank-transformed fungal biomass and bacterial density data, respectively

	Df	Sum of squares	Mean squares	*F*-value	*p*-value
Fungal biomass					
UV	1	52,415	52,415	16.427	<0.001
nTiO_2_	2	16,323	8161	2.558	0.091
Interaction	2	10,774	5387	1.688	0.199
Residuals	36	114,872	3191		
Bacterial density					
UV	1	107	106.88	0.686	0.413
nTiO_2_	2	364	182.00	1.168	0.322
Interaction	2	92	45.81	0.294	0.747
Residuals	36	5608	155.78		

Moreover, the observations of this first set of food selection experiments contradict at a first glance Feckler et al. (2015). These authors suggested that gammarids avoid leaf litter pre-exposed to UV in combination with nTiO_2_ for 24 h over those exposed to the same nTiO_2_ concentration in absence of UV. In their study, Feckler et al. (2015) intentionally avoided microbial conditioning and the UV intensity was one order of magnitude above the one assessed in the present study. Consequently, it is assumed that one of the mechanisms they suggested, namely the potential formation of harmful lignin degradation products (e.g. Prado et al. 2013), was either not initiated in the present study as a consequence of the low UV intensity or mitigated by the biofilm developing on the leaf discs. In support of this assumption, gammarids did not show any preference when given the choice between leaf discs conditioned in darkness and absence of nTiO_2_ relative to those conditioned in the presence of either 20 or 2000 μg nTiO_2_/L under similar light conditions during this study (Fig. 1b). The same observation was made when leaf discs were conditioned under UV irradiation (Fig. 1c).

Consequently, gammarids are not capable of sensing nTiO_2_ in their food that was likely adsorbed to the leaf discs, irrespective whether UV irradiation is present or not. This suggests that the uptake of these NPs is not actively avoided by these shredders, making ingestion a likely effect pathway. As this pathway has not induced any sublethal response in *G. fossarum* over 30 days at nTiO_2_ concentrations a factor of 2 above the highest concentration tested here (Lüderwald et al. 2019b), risks for this shredder species may be considered low. Nonetheless, the relevance of NP exposure through food depends on NP identity (see for silver and copper oxide NPs, Lüderwald et al. 2019b and Pradhan et al. 2012) and potentially their interaction with environmental factors.

### Microbial leaf litter decomposition

**Fig. 3:**
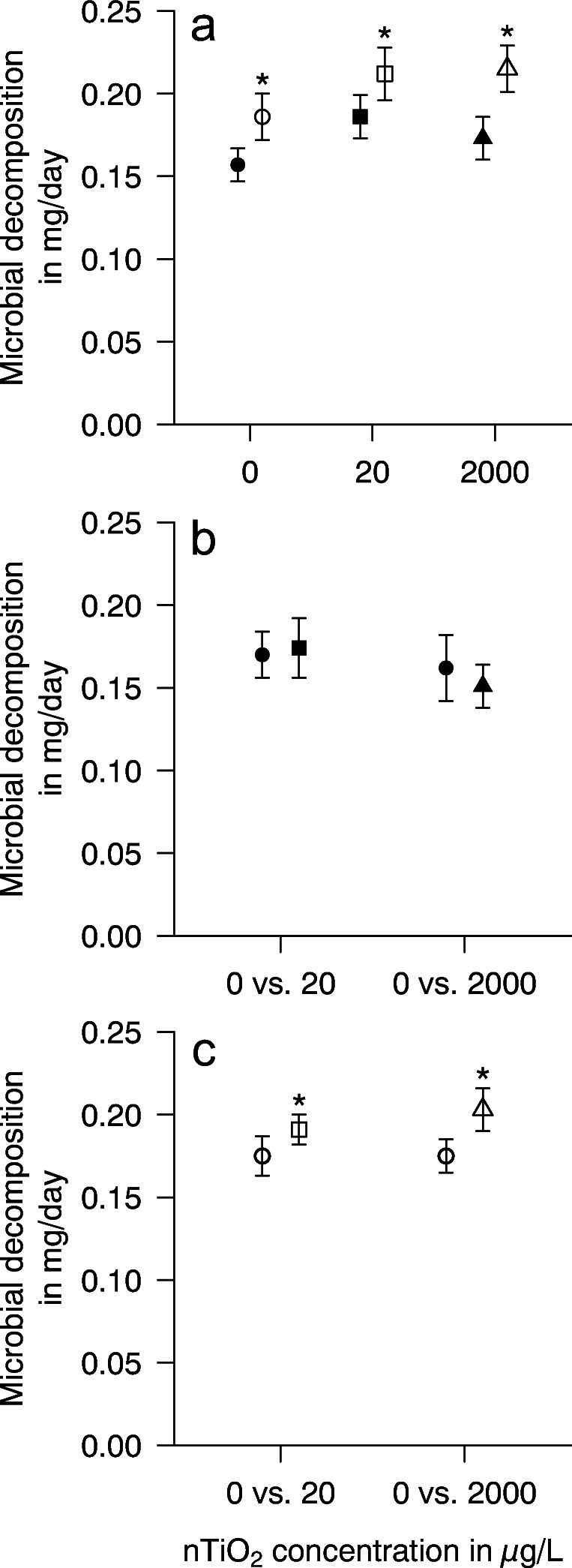
Mean (±95% CI) microbial leaf mass loss. **a** The impact of UV at a given nTiO_2_ concentration was assessed in a pairwise design; microbial leaf mass during conditioning in darkness (filled symbols) or under UV irradiation (open symbols) fully crossed with 0 (circles), 20 (squares), and 2000 (triangles) μg nTiO_2_/L, respectively (pairwise *t*-tests; *p* < 0.001; *n* ≥ 48). **b** The impact of two nTiO_2_ concentrations during conditioning in darkness was assessed in a pairwise design; microbial leaf mass during conditioning in darkness (filled symbols) in combination with 0 (circles) vs 20 (square) and 2000 (triangle) μg nTiO_2_/L, respectively (pairwise *t*-tests; *p* > 0.1; *n* ≥ 39). **c** The impact of two nTiO_2_ concentrations during conditioning under UV irradiation was assessed in a pairwise design; microbial leaf mass during conditioning under UV irradiation (open symbols) in combination with 0 (circles) vs 20 (square) and 2000 (triangle) μg nTiO_2_/L, respectively (pairwise *t*-tests; *p* > 0.01; *n* ≥ 48)

In darkness, microbial leaf decomposition, the second ecosystem-level process targeted here, is not significantly negatively affected by nTiO_2_ relative to the control (Fig. 3 a and b), while a slight tendency for stimulation could be observed. This observation contrasts earlier studies reporting at similar nTiO_2_ (up to 2000 μg/L) concentrations a reduction in microbial black alder leaf litter decomposition (Jain et al. 2019). As NP size is playing a significant role in the toxicity induced by nTiO_2_ (e.g. Seitz et al. 2014), the lower initial nTiO_2_ size employed by Jain et al. (2019) (15 vs approx. 85 nm) likely contributes to the discrepancy between studies. Two additional studies targeted the impact of nTiO_2_ on leaf decomposition in freshwater systems: the microbial decomposition of *Populus nigra* and *Ficus vasta* was reduced by up to 30%, which could be linked to changes in the leaf-associated microbial community, respiration, and enzymatic activity (Al Riyami et al. 2019; Du et al. 2018). In contrast to the present study, the concentrations used by these authors were up to two orders of magnitude (up to 500 mg/L) above those applied here. Moreover, the exposure duration exceeded 40 days. It may hence be suggested that an important factor for the insignificant impact of nTiO_2_ on the microbial leaf decomposition in darkness during the present study is related to the substantially lower concentration-time product (the product of the exposure concentration and duration) relative to the earlier publications.

In support of our hypothesis, the presence of UV at this low intensity increased leaf degradation either through abiotic processes (i.e. photodegradation) (Hunting et al. 2019) or the stimulation of microbial decomposers in the absence and presence of nTiO_2_. The effect sizes induced by UV irradiation remained among nTiO_2_ concentrations with 15–25% rather stable (Fig. 3a). By comparing the impact of 20 and 2000 μg nTiO_2_/L under UV irradiation to that of the respective control (i.e. UV irradiation in the absence of nTiO_2_), the microbial leaf decomposition showed with 10 and 16% a significant increase (Fig. 3c).

The stimulation of microbial leaf decomposition even in the absence of nTiO_2_ is contrary to an earlier study (Díaz Villanueva et al. 2005) but may be explained by UV-induced photodegradation of lignin to more easily assimilable or leachable organic carbon (King et al. 2012) as suggested in terrestrial systems (Rozema et al. 1997). As changes in the stoichiometry of the leaf litter are rather marginal (Table 1), we are not able to provide data supporting this mechanism. Another explanation would be a shift in the leaf-associated microbial community favouring microorganisms with a higher leaf decomposition efficiency. While fungal biomass and bacterial abundance did not point to changes, it is still possible that the microbial community structure and activity changed as reported for UV irradiation (Denward et al. 2001), visible light (Du et al. 2017), or artificial light at night (Pu et al. 2019).

These mechanisms may also be relevant for the treatments that were additionally exposed to the semi-conductor nTiO_2_, as ROS production is usually elevated under such conditions as shown in one of our earlier studies under similar conditions (Lüderwald et al. 2019a). Higher levels of ROS could in turn stimulate photochemical degradation of lignin and other recalcitrant organic substances. Moreover, shifts in leaf-associated communities towards ROS-tolerant species as observed in periphyton with implications in the biofilm biomass, metabolic activity (Binh et al. 2016; Wright et al. 2018), and the production of extracellular polymeric substances (Kumari et al. 2014) are likely. Consequently, and despite the fact that a final conclusion on the underlying mechanisms cannot be drawn, we suggest the interaction between photochemical and biological processes as the most likely cause of the effects in microbial leaf decomposition.

## Conclusion

The present study clearly shows that nTiO_2_ — particularly in combination with ambient UV irradiation — has the potential to impact the ecosystem function of microbial leaf litter decomposition. Although this process was positively affected, indicating a quicker incorporation of energy bound in leaves into stream food webs, this effect may indicate negative consequences for ecosystems: a faster decomposition suggests also an earlier loss of this resource from the ecosystem following leaf fall. Consequently, organisms depending on leaf litter as food or habitat may need to search for alternatives, potentially leading to competition earlier in the year and in those species’ life cycles. More importantly, the data highlighted that the multiple functions a microbial community performs — in the present study represented by microbial leaf decomposition and leaf palatability — are not necessarily equally sensitive. Consequently, focusing on only one of the many functions realized by the same microbial community may be misleading for environmental management and decision-making.

## Declarations

Ethics approval and consent to participate
